# Prognostic Value of Platelet to Lymphocyte Ratio for Myocardial Infarction: A Systematic Review and Meta‐Analysis

**DOI:** 10.1002/clc.70215

**Published:** 2025-10-14

**Authors:** Hengxu Yu, Shitao Li, Yutong Wu, Xiangpeng Ren

**Affiliations:** ^1^ School of Medicine Jiaxing University Jiaxing Zhejiang China; ^2^ First Clinical Medical College Shanxi Medical University Taiyuan Shanxi China

**Keywords:** meta‐analysis, myocardial infarction, platelet‐to‐lymphocyte ratio, prognostic value

## Abstract

**Background:**

Correlations between platelet‐to‐lymphocyte ratio (PLR) and prognosis in patients with acute myocardial infarction (AMI) are reported in more studies, though there is no evidence‐based data.

**Methods:**

Databases (PubMed, Embase, Web of Science, and Cochrane Library) were searched from their inception to April 19, 2024, to retrieve articles discussing associations between PLR and clinical outcomes in AMI patients. The primary outcomes, comprising mortality and major adverse cardiovascular events (MACE), were assessed with odds ratios (OR) and their 95% confidence intervals (CI). Sensitivity analyses and subgroup analyses were utilized to probe the results' robustness and potential heterogeneity sources. Analysis was carried out utilizing the software of Review Manager 5.4 & STATA 15.0.

**Results:**

This article selected 18 cohort studies, covering 16,545 AMI patients. The meta‐analysis found that elevated PLR was significantly linked with mortality in AMI patients (OR = 1.06; 95% CI: 1.04–1.08, *p* < 0.00001). Additionally, PLR was highly linked with MACE risks in AMI patients (OR = 1.495; 95% CI: 1.24–1.80, *p* < 0.0001). Further subgroup analyses discovered a significant correlation between PLR and mortality in prospective studies (OR = 1.07; 95% CI: 1.05–1.09), studies with a sample size ≥ 500 (OR = 1.06; 95% CI: 1.04–1.08), patients under 70 years of age (OR = 1.07; 95% CI: 1.05–1.09), studies from European regions (OR = 1.08; 95% CI: 1.06–1.10), patients with ST‐elevation myocardial infarction (OR = 1.09; 95% CI: 1.07–1.11), and those with a PLR cutoff value < 140 (OR = 1.07; 95% CI: 1.05–1.09) (*p* < 0.05). For MACE, similar subgroup analyses also proved an obvious correlation between PLR and MACE in the aforementioned subgroups (*p* < 0.05).

**Conclusion:**

PLR values are linked with mortality and MACE in AMI patients. PLR serves as an effective prognostic biomarker for AMI patients, providing precious opinions for sensible therapeutic decisions in AMI treatments.

## Summary

- PLR is significantly associated with the poor prognosis of AMI patients and can serve as an effective prognostic biomarker.
- This study included 18 studies with a total of 16,545 AMI patients, finding that elevated PLR significantly increases the risk of mortality.
- PLR is significantly associated with the risk of MACE in AMI patients.

## Graphical abstract

Background: The prognostic value of platelet‐to‐lymphocyte ratio (PLR) in acute myocardial infarction (AMI) remains inconsistent.

Finding: This meta‐analysis of 18 studies shows higher PLR significantly predicts increased mortality and major adverse cardiovascular events.

Conclusion: PLR serves as a potent and accessible prognostic biomarker for risk stratification in AMI patients.

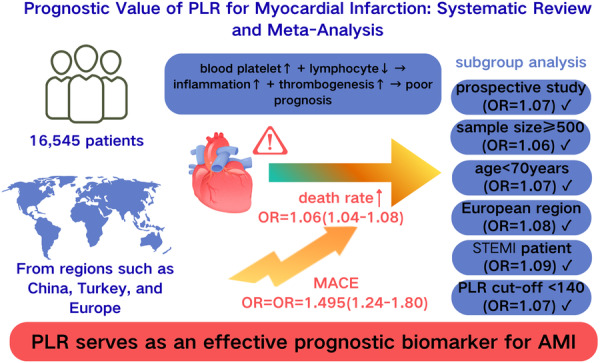

## 1 Introduction

Acute myocardial infarction (AMI) represents a significant global health concern [1]. The global incidence of AMI varies among different age groups [2], with 3.8% in people under 60 years old and 9.5% in people over 60 years old [3]. At the same time [3], AMI is one of the leading causes of death globally [2, 4]. In 2019, around 8.9 million people died of AMI worldwide [5], accounting for 16% of the total global deaths. In 2021, about 9 million died of AMI worldwide [6]. The incidence and mortality of AMI differ significantly among different regions [7], with the highest rates observed in Eastern and Central Europe, and relatively low rates in Africa and Asia. At present, young and middle‐aged people, older people, and people with genetic heart diseases have higher risks of AMI [8]. Hypertension, hyperlipidemia, hyperglycemia, bad living habits [9], and poor psychological factors are also risk factors for AMI. It is expected that the global incidence of AMI will increase from 1655/100,000 people in 2017 to 1845/100,000 in 2030. Besides, population aging contributes to the increase in the absolute number of AMI deaths.

Although the incidence of AMI has declined during recent decades, it still poses a global health threat, particularly in Asia. In recent years, with the popularization of laboratory tests [4], the prognostic value of laboratory test results on AMI patients has undergone a revolutionary change [7]. AMI is caused by total coronary artery occlusion, with a high mortality rate [4]. In recent years, as an emerging biomarker, the platelet‐to‐lymphocyte ratio (PLR) has gradually attracted attention. This biomarker is used to evaluate the prognosis of AMI. For example, Ahmet Temiz et al. discovered a higher hospital mortality rate in AMI patients with PLR > 144 (12.7% vs. 5.9%, *p* = 0.004); the multivariate analysis confirmed that PLR was an independent predictor for in‐hospital cardiovascular death (hazard ratio (HR): 2.16, 95% confidence interval (CI): 1.16–4.0, *p* = 0.014). PLR is an important prognostic marker in AMI patients, and higher PLR values are significantly associated with a higher risk of in‐hospital mortality in AMI patients. PLR plays a crucial role in predicting survival outcomes in AMI patients. However, the predictive performance of PLR in AMI remains to be further explored.

Weng Li [10], in a meta‐analysis published in 2016, appraised the prognostic value of platelet‐lymphocyte ratio (PLR) in acute coronary syndrome (ACS) and demonstrated notably higher risks of poor in‐hospital and long‐term outcomes in patients with elevated PLR. However, their meta‐analysis primarily included studies carried out in Europe, possibly compromising the representativeness of results. Furthermore, they did not perform subgroup analyses on all indicators based on factors like lesion type, which may limit the accuracy. Besides, after its publication, nine new related studies [1, 2, 4, 5, 6, 11, 12, 13, 14] have been published, and their conclusions are inconsistent. Therefore, our purpose is to incorporate recently published clinical data to conduct a comprehensive meta‐analysis based on previous studies to appraise the prognostic value of PLR for AMI individuals and explore possible influencing factors.

## 2 Methods

### 2.1 Literature Search

The reports of this study followed the guidelines outlined in the Preferred Reporting Items for Systematic Reviews and Meta‐Analyses (PRISMA2020) Statement. The study protocol has been registered in the International Prospective Register of Systematic Reviews (PROSPERO: CRD42025609543). Two investigators (Hengxu Yu and Shitao Li) were responsible for formulating search strategies. Multiple databases such as PubMed, Embase, Web of Science, and Cochrane Library were searched from the establishment of the database to April 19, 2024. A wide range of terms were used for search, including keywords like platelet‐to‐lymphocyte ratio and AMI (Figure S1).

### 2.2 Study Selection

Inclusion criteria: (1) patients diagnosed with AMI by a combination of electrocardiogram (ECG) and computed tomography angiography (CTA); (2) studies focusing on assessing the prognostic impact of PLR on major adverse cardiovascular events (MACE); (3) studies recorded odds ratio, risk ratio, and hazard ratio with their 95% CI data or mean and standard deviation.

Exclusion criteria: (1) reviews, comments, meeting summaries, case reports, and letters; (2) studies lacked sufficient information to calculate odds ratios (OR), risk ratio (RR), HR, or standardized mean difference (SMD) and 95% CI; (3) studies that reported no survival data; (4) studies with duplicate or overlapping data.

Two researchers (Hengxu Yu and Shitao Li) separately reviewed study titles and abstracts retrieved from the database, downloaded and read full‐text articles to determine eligible studies. Any differences would be resolved through consensus.

### 2.3 Data Extraction

Two researchers (Hengxu Yu and Shitao Li) independently extracted data. Any differences would be settled through the consensus of all co‐authors. The extracted data comprised the name of the first author, year of publication, country (location), type, sample size, patient age, duration, treatment method, specific immune checkpoint inhibitors, testing time, cut‐off value, follow‐up period, and outcome indicators. For studies reporting lymphocyte‐to‐platelet ratio (LPR) data [5, 15], we took the relevant OR value and the reciprocal of the corresponding CI, reversed upper and lower confidence limits, and converted the LPR into a PLR value for statistical analysis.

### 2.4 Quality Assessment

The Newcastle‐Ottawa Scale (NOS) was applied to assess the quality of the included studies from three domains (selection, comparability, and results), with a maximum score of 9 [16]. Studies with scores of 7–9 were rated as high quality.

### 2.5 Statistical Analysis

The pooled OR or SMD and relative 95% CI were computed to assess the prognostic value of PLR in AMI patients. Heterogeneity was evaluated using Cochran's Q test and Higgins I‐squared (*I* ^2^) statistic [15]. *I* ^2^ > 50% or *p* < 0.1 represented significant heterogeneity. A random‐effects model was utilized to analyze all data. Furthermore, subgroup and sensitivity analyses were performed to verify the robustness of the results and explore potential sources of heterogeneity. Funnel plots and Egger's test were utilized to assess publication bias. *p* < 0.05 was considered statistically significant. STATA 15.0 and ReviewManager 5.4 were applied for all statistical analyses.

### 2.6 Declaration the Use of Any Artificial Intelligence Generated Content (AIGC) Tools

We declare that we did not use any Artificial Intelligence Generated Content (AIGC) tools.

## 3 Results

### 3.1 Research Characteristics

46 articles were obtained from databases, and 3 duplicates were deleted. After reviewing the titles and abstracts of the remaining studies, 2 unrelated studies were eliminated. The full texts of 41 studies were then evaluated, and 2 of these studies were excluded mainly due to insufficient relevant data required for survival analysis. Finally, this meta‐analysis included 18 studies with a total of 16,545 patients [5, 6, 7, 10, 12, 13, 14, 15, 16, 17, 18, 19, 20, 21, 22, 23, 24, 25] (Table S1).

Of the 18 eligible studies, 8 studies were conducted in Turkey, 1 study in Poland, 1 study in France, 1 study in the United States, 1 study in Australia, and the remaining 6 studies in China. All studies adopted a controlled method and categorized participants into high‐PLR and low‐PLR groups. Regarding PLR measurements, six studies reported PLR measured at baseline, one study reported PLR after treatment, and one study reported PLR at both baseline and after treatment. In terms of PLR evaluation, 8 studies discussed the prognostic significance of PLR on AMI, and 7 studies discussed the prognostic value of PLR on AMI (Table 1). As some studies reported multiple parallel control groups simultaneously, this study ultimately extracted 24 control groups.

**Table 1:** Data extraction figure from literature.

Author yellow mean＋range white mean＋SD blue mean＋SE orange median＋IQR gray median (min–max)	Study period	Region	Study design	Population	No. of patients	Gender		Mean age	PLR cut‐off	NOS Score
						Male	Female			
Ahmet 2014	2009.1–2011.11	Turkey	Retrospective study	STEMI	636	505	131	High:63.7/Low:61.4	144	7
Bartosz 2017	2016.1–2017.1	Poland	Prospective study	STEMI	523	217	306	64	110	6
Handan 2014		Turkey	Prospective study	CVD	424	250	174	50.36	108.56	8
Fatma 2023	2017.1–2021.12	Turkey	Prospective study	ACS	1103	759	344	68.2		7
Qian Zhang 2020	2017.1–2019.12	China	Retrospective study	STEMI	217	173	44	61.01	118.07	8
Oktay 2021	2016.12–2020.10	Turkey	Retrospective study	STEMI	247	185	62	58.06	144	7
Nicolas 2018A	2015.1–2017.12	France	Retrospective study	ICA	270	178	92	g1:69.4/g2:72.1/g3:73.8/g4:75.1	86.6	8
Nicolas 2018B	2015.1–2017.12	France	Retrospective study	ICA	270	178	92	g1:69.4/g2:72.1/g3:73.8/g4:75.1	111.7	
Nicolas 2018C	2015.1–2017.12	France	Retrospective study	ICA	270	178	92	g1:69.4/g2:72.1/g3:73.8/g4:75.1	148.3	
Jianlong Sheng 2021	2018.7–2019.9	China	Retrospective study	ACS	205	138	67	UA:63.6/non:66.5/ST:71.1	107.01	7
Abdulkadir 2014		Turkey	Retrospective study	STEMI	319	213	106	60.8	160	7
Basem 2012A	2004.9–2006.12	USA	Prospective study	NSTEMI	619	417	202	low:61.1/mid:64.1/high:68.3	176	8
Basem 2012B										
Alparslan 2014	2012.6–2013.9	Turkey	Prospective study	STEMI	520			60	126	7
Cuneyt 2015A		Turkey	Retrospective study	STEMI	304	246	58	low:60.0/mid:60.0/high:59.4	201	8
Cuneyt 2015B										
Zhongyuan Meng 2021		China	Retrospective study	NSTEMI	1273	707	566	low:71.9/high:74.2	195.8	8
Li Li 2020	2012.1–2016.2	China	Retrospective study	AMI	1001	805	196	young:49.7/old:67.3	147	7
Yan Chen 2023	2015.12–2021.12	China	Retrospective study	AMI	1550	926	624	73.1	187.6	9
Xi‐peng Sun	2005.1–2010.12	China	Retrospective study	STEMI	5719	4414	1305	59.2	127.5	6
Mustafa 2014A	2012.1–2013.8	Turkey	Retrospective study	ACS	587	401	186	61.8	142	8
Mustafa 2014B										
Lee 2018A	2009.10–2013.5	Australia	Retrospective study	CAD	514	354	160	64	137	8
Lee 2018B	2009.10–2013.5	Australia	Retrospective study	CAD	514	354	160	64	137	

### 3.2 Research Quality

One article had a NOS score of 9 points; 8 articles had a NOS score of 8 points; 7 articles had a NOS score of 7 points; and 2 articles had a NOS score of 6 points (Tables S1–S2).

### 3.3 Results of Meta‐Analysis

#### 3.3.1 PLR and Mortality

**Figure 1:**
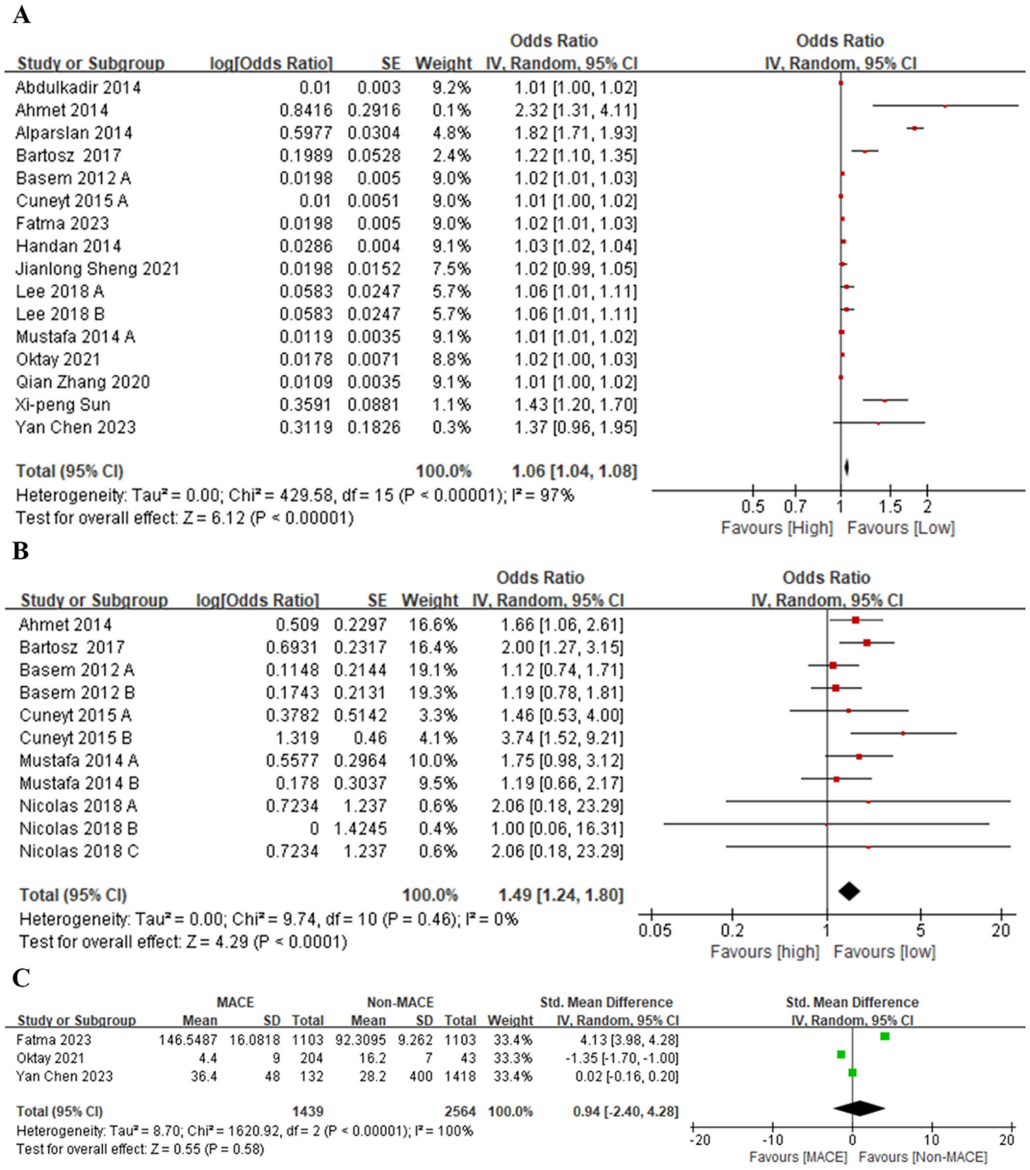
(A) Odds ratio for PLR and mortality; (B) Odds ratio of PLR and MACE (categorical variables); (C) Odds ratio of PLR and MACE (continuous variables).

Data from 16 studies were systematically pooled utilizing a random‐effects model. The results showed that PLR was positively associated with the risk of mortality. The pooled OR was 1.06 (95% CI: 1.04, 1.08), which was statistically significant (*Z* = 6.12, *p* < 0.00001), indicating that PLR may be a valid indicator for predicting the risk of mortality (Figure 1A).

#### 3.3.2 PLR and MACE

##### 3.3.2.1 Categorical Variables

7 studies reported data on PLR as categorical variables for MACE in AMI. It was found that the pooled OR value was 1.49 (95% CI: 1.24, 1.80), indicating that as PLR increased, the risk of MACE also increased (Figure 1B).

##### 3.3.2.2 Continuous Variables

3 studies reported data on PLR as continuous variables for MACE in AMI [7, 11, 15]. The results disclosed that the PLR was significantly higher in patients with MACE than those without MACE (SMD = 0.94, 95% CI: −0.24, 4.28; *p* < 0.00001), with no substantial heterogeneity (*I* ^2^ = 0%, *p* = 0.90) (Figure 1C).

### 3.4 Sensitivity Analysis

**Figure 2:**
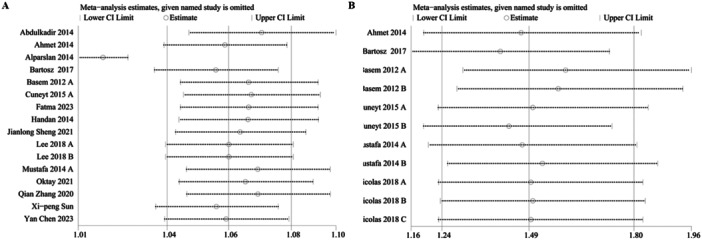
(A) Sensitivity analysis of PLR and mortality; (B) Sensitivity analysis of PLR and MACE.

Sensitivity analyses were carried out on mortality and MACE to test the robustness of the results. The results indicated that the effect size remained consistent within the original range after deleting each study sequentially. This proved that the predictive performance of PLR for mortality (Figure 2A) and MACE (Figure 2B) was not disproportionately affected by any particular study, thus validating the robustness of the results.

### 3.5 Publication Bias

**Figure 3:**
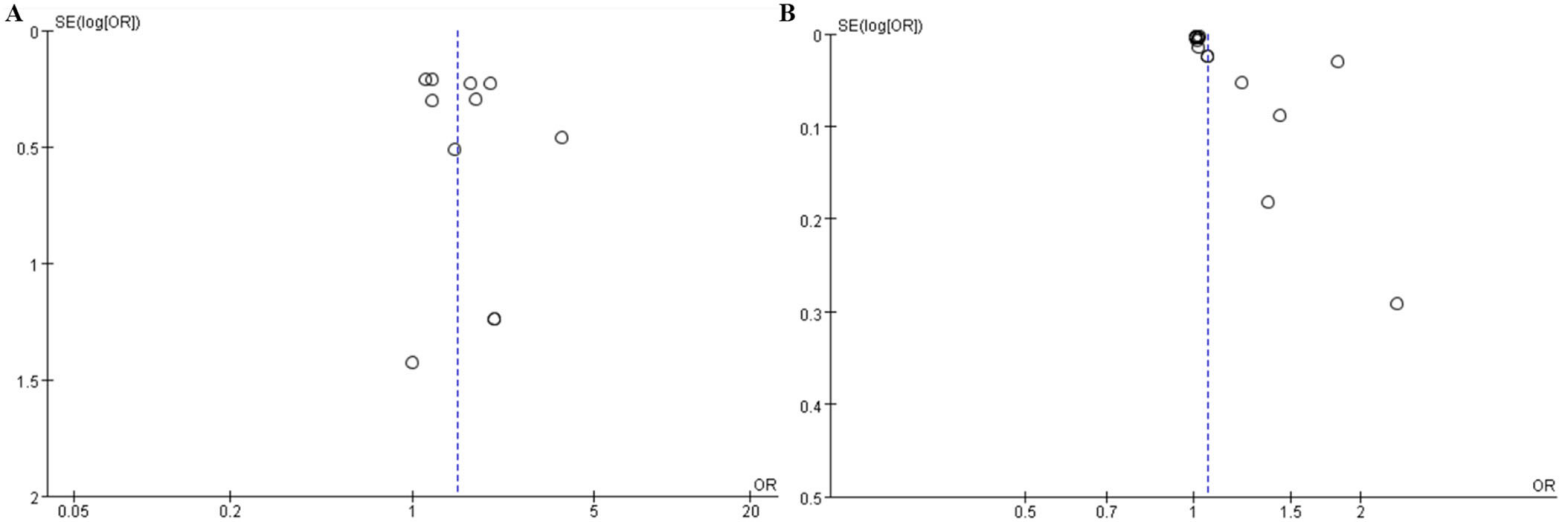
(A) Funnel plot for publication bias assessment of MACE; (B) Funnel plot for publication bias assessment of mortality.

**Figure 4:**
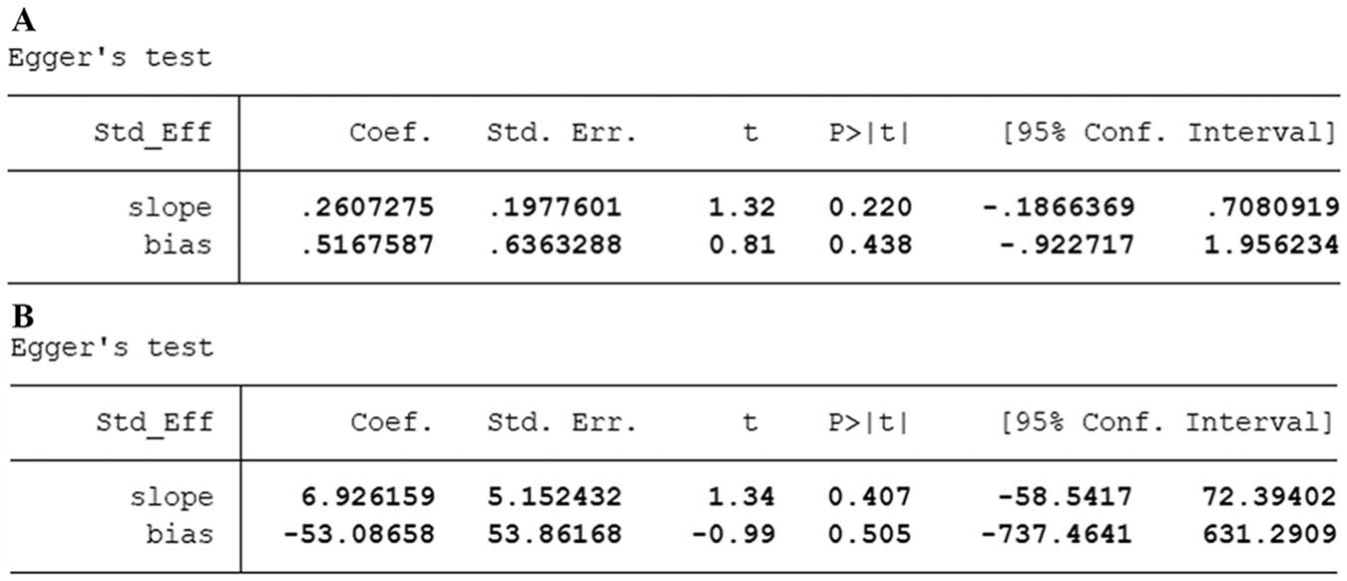
(A) Egger′s test for mortality; (B) Egger′s test for MACE.

The funnel plot showed that no publication bias was noted for MACE (Figure 3A), while possible publication bias was observed for mortality (Figure 3B). Additionally, Egger's test confirmed that publication bias may exist for mortality (Figure 4A) (*p* = 0.016), but no publication bias for MACE (*p* = 0.438) (Figure 4B).

### 3.6 Subgroup Analysis

Subgroup analysis was carried out for mortality and MACE based on study designs, sample sizes, age, region, population type, and cut‐off of PLR. In terms of mortality, there were significant differences in PLR among prospective studies, sample sizes ≥ 500, age < 70 years, European region, STEMI population, and PLR cut‐off values < 140 (*p* < 0.05), yet no significant differences were found in other subgroups. For MACE, PLR also showed significant differences in prospective studies, sample sizes ≥ 500, age < 70 years, European region, STEMI population, and PLR cut‐off < 140 (*p* < 0.05), and no significant differences were noticed in other subgroups (Table 2).

**Table 2:** Subgroup analysis.

Subgroup	Mortality				MACE			
	Study	OR [95% CI]	*p* value	*I* ^2^	Study	OR [95% CI]	*p* value	*I* ^2^
**Total**	16	1.06 [1.04–1.08]	< 0.00001	97%	4	0.38 [0.16–0.90]	0.03	0%
Study design								
Prospective	5	1.16 [1.10–1.23]	< 0.00001	99%	3	1.37 [0.97–1.95]	0.14	50%
Retrospective	11	1.01 [1.01–1.02]	0.0001	71%	9	1.26 [0.49–3.26]	< 0.00001	93%
Sample size								
≥ 500	10	1.15 [1.10, 1.21]	< 0.00001	98%	7	1.05 [0.48, 2.27]	0.9	95%
< 500	6	1.02 [1.01, 1.02]	< 0.00001	70%	5	2.31 [1.25, 4.24]	0.007	0%
Mean/median age								
≥ 70 y	2	1.02 [0.99–1.05]	0.11	61%	4	0.68 [0.13–3.68]	0.02	69%
< 70 y	14	1.02 [1.01–1.02]	< 0.00001	97%	8	1.52 [1.21–1.89]	0.22	27%
Region								
Asia	4	1.05 [1.00, 1.12]	0.07	84%	1	0.16 [0.11, 0.23]	< 0.00001	NA
Europe	9	1.08 [1.05, 1.11]	< 0.00001	98%	9	1.76 [1.39, 2.22]	< 0.00001	0%
America	1	1.02 [1.01, 1.03]	< 0.00001	NA	2	1.16 [0.86, 1.55]	0.34	0%
Oceania	2	1.06 [1.02, 1.10]	0.0008	0%	0			
Population								
STEMI	9	1.14 [1.09, 1.18]	< 0.00001	98%	4	1.93 [1.44, 2.57]	< 0.00001	0%
NSTEMI	1	1.02 [1.01, 1.03]	< 0.00001	NA	3	0.60 [0.15, 2.34]	0.46	97%
Unclassified	6	1.02 [1.01, 1.03]	< 0.00001	66%	5	1.47 [0.98, 2.19]	0.06	0%
PLR cut‐off								
≥ 140	7	1.01 [1.01–1.02]	< 0.00001	58%	6	1.00 [0.35, 2.83]	1	95%
< 140	8	1.15 [1.05, 1.21]	< 0.00001	98%	3	1.97 [1.27, 3.06]	0.003	0%

## 4 Discussion

In recent years, with the popularization of laboratory tests, the evaluation of the prognostic value of laboratory test results in AMI patients has undergone a revolutionary transformation [20, 21, 22]. As an emerging biomarker, PLR has gradually gained traction. PLR not only reflects the inflammatory state but also participates in thrombosis, contributing to the pathogenesis of AMI. Studies have verified that PLR may help identify individuals with high‐risk mortality among AMI patients [23]. However, the results of existing studies are controversial, and some studies do not find associations between PLR and adverse events in AMI patients. This meta‐analysis aimed to disclose correlations between PLR and the risk of MACE in individuals with AMI [24].

This study comprehensively included 18 cohort studies involving 16,545 patients. The result suggested that increased PLR levels were significantly associated with the risk of mortality and MACE in patients with AMI, confirmed by the sensitivity analysis. The results support PLR as an efficient marker in assessing the prognosis of AMI patients, consistent with previous studies [19, 26]. These studies underscore the key role of inflammation in the development and prognosis of AMI, and PLR serves as a simple hematologic indicator to help identify high‐risk patients. In addition to PLR, other hematologic biomarkers are reported to be associated with myocardial infarction. The neutrophil‐to‐lymphocyte ratio (NLR) is a hematologic inflammatory marker calculated as the absolute count of neutrophils divided by the absolute count of lymphocytes, reflecting the balance between innate inflammatory activation and adaptive immune regulation. Numerous studies have demonstrated that an elevated NLR is significantly associated with increased risks of mortality and MACE in patients with AMI, underscoring its role as a robust prognostic biomarker. Similar to PLR, both NLR and PLR leverage components of the complete blood count to quantify systemic inflammation and immune dysregulation, and both have been independently associated with adverse outcomes across various AMI subtypes. NLR emphasizes granulocyte‐driven inflammation and stress response. In contrast, PLR incorporates platelet activity and may more directly reflect prothrombotic mechanisms, particularly in ST‐elevation myocardial infarction (STEMI), where microvascular thrombosis plays a critical role.

Compared with the meta‐analysis by Li et al. [10] this study further certified the potential role of PLR in evaluating the prognosis of AMI individuals. Li et al. have demonstrated that PLR is a promising biomarker for poor prognosis in ACS patients [25, 27], consistent with our findings. However, our study further confirmed a significant association between PLR and MACE in AMI through a larger sample size and more comprehensive analysis [28, 29]. Then, our study is different from that of Li et al. in many aspects. First, our study included more studies and patients, providing stronger evidence to support the effectiveness of PLR as a prognostic assessment tool. Secondly, this study discussed the performance of PLR in different subgroups and found a significant association between PLR and MACE, while Li et al. did not perform subgroup analysis [30]. In addition, this study assessed the performance of PLR at different cut‐off values, offering more specific guidance for clinical applications.

As a novel biomarker, PLR has been demonstrated to be effective in the evaluation of prognosis in patients with AMI. PLR reflects the interaction between platelets and lymphocytes, both of which play an irreplaceable role in evaluating the prognosis of AMI patients [29, 31]. Platelets affect the development of AMI by promoting local thrombosis and mediating the inflammatory response [32, 33]. In addition, lymphocytes, as an important part of the immune system, are also crucial in regulating immune responses and maintaining immune homeostasis [16, 34, 35]. Therefore, an increase in PLR may indicate a functional imbalance between platelets and lymphocytes, which may adversely affect the prognosis of patients with AMI. Specifically, an increase in PLR may indicate an increase in inflammation levels and a disorder in immune status in patients, which in turn affects the prognosis of AMI [28, 36, 37]. To sum up, PLR, as a simple and easy‐to‐measure biomarker, has important clinical significance in appraising the prognosis of AMI individuals [15, 34, 38]. Future research should further explore the application of PLR in the prognostic assessment of AMI.

This systematic review shows stronger evidence for PLR as an effective biomarker for prognosis assessment of AMI patients and provides valuable insights for clinical decision‐making. This study not only focused on the overall correlation between PLR and adverse events in AMI patients but also explored differences in the performance of PLR in different subgroups (such as different study designs, sample sizes, ages, regions, population types, and PLR cut‐off values). In addition, publication bias was assessed to ensure the robustness of results. Based on these comprehensive analyses, the study revealed the value of PLR in evaluating the prognosis of AMI patients under different conditions and provided instructions for future clinical practice and research.

Furthermore, given its strong prognostic performance, PLR may be integrated into existing risk stratification models for AMI, such as the GRACE (Global Registry of Acute Coronary Events) or TIMI (Thrombolysis in Myocardial Infarction) scores [23]. Combining PLR with these established models could enhance their predictive accuracy for mortality and MACE, providing a more comprehensive assessment tool for clinicians to identify high‐risk patients earlier and tailor more aggressive management strategies accordingly. Future studies should focus on developing and validating a unified prognostic framework that incorporates PLR and traditional risk factors and biomarkers, evaluating its incremental value through statistical measures like net reclassification improvement (NRI) and integrated discrimination improvement (IDI).

This study also has limitations, including heterogeneity in included studies, potential publication bias, and inconsistency in the measurement methods of PLR. In addition, the relationship between PLR and the prognosis of AMI may be influenced by multiple unmeasured factors, such as the patient's original health status, treatment strategies, and lifestyle. Further research should explore the biological mechanism of PLR and assess its potential value in clinical practice. In our study, publication bias was identified for mortality (*p* = 0.016), as indicated by the funnel plot and Egger's test (Figures 3B and 4A). This bias suggests that smaller studies with negative or nonsignificant results may be underrepresented in our analysis. The potential implications of this bias on clinical interpretation are noteworthy. Specifically, the presence of publication bias may lead to an overestimation of the strength of the association between PLR and mortality in AMI patients. This overestimation could result in clinicians placing undue emphasis on PLR as a prognostic marker, potentially at the expense of other established risk factors or biomarkers. Hence, clinicians should consider the possibility of publication bias when interpreting our findings and integrate PLR into their prognostic assessments. Future research should include a broader range of studies, particularly those with negative results, to more comprehensively validate the prognostic value of PLR in AMI. PLR may help clinicians identify and manage high‐risk patients, thereby improving their treatment outcomes.

## 5 Conclusion

Our results support PLR as a potent method for evaluating the prognosis of AMI patients. Future research should further explore the biological mechanism of PLR and evaluate its potential value in clinical practice. Deeply understanding the correlations between PLR and AMI prognosis will help us spot and manage high‐risk patients more accurately, thus upgrading their treatment outcomes.

## Author Contributions

**Hengxu Yu:** conceptualization, methodology, software, funding acquisition, validation. **Shitao Li:** formal‐analysis, investigation, resources, writing – original draft, data‐curation. **Yutong Wu:** writing – original draft, writing – review and editing, visualization, supervision, project administration. **Xiangpeng Ren:** project administration, conceptualization, software, funding acquisition.

## Ethics Statement

The authors have nothing to report.

## Consent

The authors have nothing to report.

## Conflicts of Interest

The authors declare no conflicts of interest.

## Supporting information


**Figure S1:** Literature retrieval strategy.

**Table S1:** NOS score for study quality.

## Data Availability

The datasets generated during and/or analyzed during the current study are available from the corresponding author on reasonable request.
