# Supplementary material for: Prognostic Value of Platelet to Lymphocyte Ratio for Myocardial Infarction: A Systematic Review and Meta‐Analysis
Source: Clin Cardiol. 2025 Oct 14;48(10):e70215. doi: 10.1002/clc.70215 (PMC12520137; doi:10.1002/clc.70215)
Supplement: Supplementary file 1 — Figure S1: Literature retrieval strategy. [file CLC-48-e70215-s001.docx]

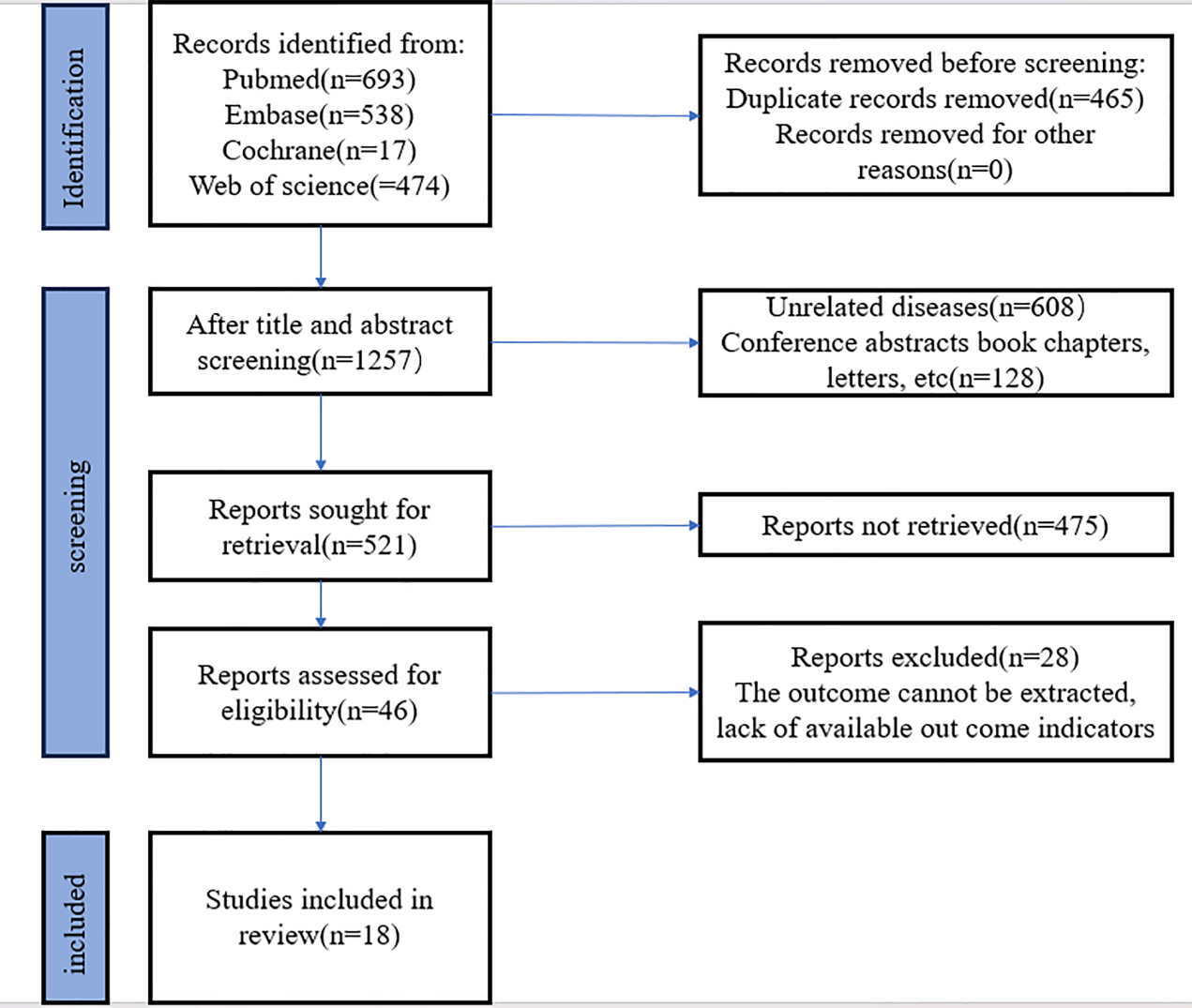


**Figure S1:** Literature retrieval strategy

**Table S1:** NOS score for study quality

(A)Quality evaluation of the eligible studies with Newcastle–Ottawa scale.

| **Study** | **Selection** | | | | **Comparability** | | **Outcome** | | |
| --- | --- | --- | --- | --- | --- | --- | --- | --- | --- |
|  | **Representativeness** | **Selection of**  **non-exposed** | **Ascertainment**  **of exposure** | **Outcome not present at start** | **Comparability on most important factors** | **Comparability on other risk factors** | **Assessment of outcome** | **Long enough follow-up (median≥1 year)** | **Adequacy**  **(completeness) of follow-up** |
| Alparslan Kurtul et al. | * | * | * | * | * | - | * | - | * |
| Basem Azab et al. | * | * | * | * | * | * | * | - | * |
| Elshaimaa Seaouda et al. | * | * | * | * | * | - | * | * | * |
| AcarWenzhang Li et al. | * | * | * | * | * | * | * | * | * |
| Handan Inonu Koseoglu et al. | * | * | * | * | * | - | * | * | * |
| Fatma Özpamuk Karadeniz et al. | * | * | * | * | * | - | * | * | * |

*indicates criterion met; - indicates significant of criterion not met.

**(B)** Quality evaluation of the eligible studies with Newcastle–Ottawa scale

| **Study** | **Selection** | | | | **Comparability** | | **Outcome** | | |
| --- | --- | --- | --- | --- | --- | --- | --- | --- | --- |
|  | **[Appropriateness](javascript:;)** | **Representativeness** | **Contrast selection** | **Determination of contrast** | **Comparability on most important factors** | **Comparability on other risk factors** | **Identification of exposure factors** | **Exposure factors were determined by the same method** | **Nonresponse rate** |
| Ahmet Temiz  et al. | * | * | * | * | * | - | * | * | - |
| Bartosz Hudzik et al. | * | * | * | * | - | - | * | * | - |
| Erdal Durmus et al. | * | * | * | * | * | - | * | * | * |
| Lee et al. | * | * | * | * | * | - | * | * | - |
| Mustafa Oylumlu et al. | * | * | * | * | * | - | * | * | * |
| Xi-peng Sun et al. | * | * | * | * | * | - | * | * | - |
| Yan Chen et al. | * | * | * | * | * | - | * | * | * |
| Zhongyuan Meng et al. | * | * | * | * | * | - | * | - | * |
| Hongling et al. | * | * | * | * | * | - | - | * | * |
| LILI et al. | * | * | * | * | * | - | * | * | * |
| zhongyang et al. | - | * | * | * | * | - | * | * | * |
| Cuneyt et al. | * | * | * | * | * | - | * | * | * |
| Abdulkadir Yildiz et al. | * | * | * | * | * | - | * | * | * |
| Jianlong Sheng et al. | * | * | * | * | - | - | * | * | * |
| Nicolas Massiot et al. | * | * | * | * | * | * | * | * | * |
| Oktay ŞENÖZ et al. | * | * | * | * | - | - | * | * | - |
| Qian Zhang et al. | * | * | * | * | * | - | * | * | * |
| et al. | * | * | * | * | * | - | * | * | * |

*indicates criterion met; - indicates significant of criterion not met.
